# Sex difference in frontal plane hip moment in response to lateral trunk obliquity during single-leg landing

**DOI:** 10.1186/s13102-022-00460-y

**Published:** 2022-04-15

**Authors:** Shohei Taniguchi, Tomoya Ishida, Masanori Yamanaka, Ryo Ueno, Ryohei Ikuta, Masato Chijimatsu, Mina Samukawa, Yuta Koshino, Satoshi Kasahara, Harukazu Tohyama

**Affiliations:** 1 Faculty of Health Sciences, Hokkaido University, West 5, North 12, Kitaku, Sapporo, 060-0812 Japan; 2 Rehabilitation Center, NTT Medical Center Sapporo, Sapporo, Japan; 3 Faculty of Health Science, Hokkaido Chitose College of Rehabilitation, Chitose, Japan

**Keywords:** Anterior cruciate ligament, Prevention, Gender difference, Knee valgus, Pelvis, Biomechanics

## Abstract

**Background:**

Lateral trunk obliquity during landing is a characteristic of anterior cruciate ligament (ACL) injuries in female athletes and affects their knee and hip kinetics and kinematics. However, it is unclear whether these effects differ between females and males. The purpose of this study was to compare the effects of lateral trunk obliquity on knee and hip kinetics and kinematics in females and males during single-leg landing.

**Methods:**

Eighteen female (aged 22.1 ± 1.5 years) and 18 male participants (aged 21.8 ± 1.1 years) performed single-leg landings under two conditions: (1) without any instructions about trunk position (natural) and (2) with leaning their trunks laterally 15° from the vertical line (trunk obliquity). The kinetics and kinematics of their hip and knee were analyzed using a three-dimensional motion analysis with a force plate. Two-way repeated-measures ANOVA (sex × trunk obliquity) and Bonferroni pairwise comparisons were conducted.

**Results:**

The trunk obliquity angle at initial contact was significantly greater in the trunk-obliquity landing condition than in the natural landing condition (natural 4.0 ± 2.2°, trunk-obliquity 15.1 ± 3.6°, *P* < 0.001) with no sex difference (95% CI − 1.2 to 2.2°, *P* = 0.555). The peak knee abduction moment was significantly larger in the trunk-obliquity landing condition than in the natural landing condition (trunk-obliquity, 0.09 ± 0.07 Nm/kg/m; natural, 0.04 ± 0.06 Nm/kg/m; *P* < 0.001), though there was no sex or interaction effect. A significant interaction between sex and landing condition was found for the peak hip abduction moment (*P* = 0.021). Males showed a significantly larger peak hip abduction moment in the trunk-obliquity landing condition than in the natural landing condition (95% CI 0.05 to 0.13 Nm/kg/m, *P* < 0.001), while females showed no difference in the peak hip abduction moment between the two landing conditions (95% CI − 0.02 to 0.06 Nm/kg/m, *P* = 0.355).

**Conclusions:**

The knee abduction moment increased with a laterally inclined trunk for both female and male participants, while the hip abduction moment increased in males but not in females. It may be beneficial for females to focus on frontal plane hip joint control under lateral trunk-obliquity conditions during single-leg landing.

## Background

An anterior cruciate ligament (ACL) injury is a severe sports injury. Approximately 70% of ACL injuries occur in noncontact situations, including cutting, pivoting, or single-leg landing [1]. Female athletes have a greater risk of noncontact ACL injury than male athletes [2]. The injury rate in female athletes remained higher than that in male athletes over the past decade [2]. Although various ACL prevention studies have been conducted, the need for improved ACL injury prevention methods for female athletes remains high. Large external knee abduction moment is caused high ACL strain during cadaveric single-leg landing simulations [3–5]. The external knee abduction moment during landing was a predictive factor of ACL injury in female athletes [6]. Therefore, jump-landing training to prevent ACL injury in female athletes has focused on reducing knee abduction moment [7].

Lateral trunk obliquity toward the landing leg increases the external knee abduction moment and knee abduction angle while decreasing the hip adduction moment or increasing the hip abduction moment [8–15]. On the other hand, lateral trunk obliquity showed no effect on knee flexion moments [9] and no effect on or decreased knee internal rotation moments [9, 10, 12]. Changes in the center-of-mass position and distribution by trunk motion affect the external loading on the knee and hip [11, 16]. The kinetic chain between trunk motion and hip and knee motion is also suggested as the mechanism [13, 17]. Furthermore, a video analysis study showed that females who sustained ACL injuries demonstrated 11.1° of lateral trunk obliquity toward the injured leg at the time of injury, whereas males demonstrated − 5.5° of lateral trunk obliquity [18]. In other video analysis studies on ACL injuries sustained by professional soccer players, females showed 15° of lateral trunk obliquity toward the injured leg, whereas males showed 5° of lateral trunk obliquity [19, 20]. Another report on ACL injuries of professional female netball players showed that 44% of studied individuals demonstrated lateral trunk obliquity toward the injured knee [21]. Therefore, the prevention of ACL injuries in female athletes has recently emphasized the importance of neuromuscular control of the trunk to avoid lateral trunk obliquity, thus reducing the knee abduction moment and angle [9, 22–24]. However, ACL injuries do not occur during landing or cutting tasks in laboratory motion analysis studies, even though participants display a lateral incline of their trunk that is at an angle similar to or greater than that observed in video analysis studies where ACL injuries do occur [10, 12, 14, 15]. A previous study showed that the lateral trunk obliquity angle was 17.0° for healthy females and males at the time of ground contact during double-leg landing with mid-flight lateral trunk bending [14]. In addition, another study on shuttle-run cutting reported that males showed a larger lateral trunk inclination toward the cutting leg than females, while there was no difference in knee abduction angles between the sexes [25]. Therefore, frontal plane knee and hip joint controls during landing with lateral trunk obliquity are important to prevent ACL injuries in addition to the avoidance of lateral trunk obliquity. Control of the frontal plane hip joint contributes to decreasing knee abduction and lateral trunk obliquity via the pelvis [11, 26]. Sex differences in the impact of lateral trunk obliquity on frontal plane knee and hip joint control may be one of the reasons lateral trunk obliquity is observed only in females who sustain ACL injuries. However, no study has examined the effect of lateral trunk obliquity on frontal plane knee and hip moments and angles in females and males during landing.

The purpose of the present study was to compare the effects of lateral trunk obliquity on frontal plane knee and hip moments and angles between females and males during single-leg landing. The hypotheses were that lateral trunk obliquity would increase the knee abduction moment, knee abduction angle and hip abduction moment in participants. Furthermore, female participants were expected to show a greater increase in knee abduction moment due to lateral trunk obliquity during landing than male participants. Additionally, we confirmed knee and hip moments and angles in the sagittal and horizontal planes because ACL injuries are caused by multiplanar loading mechanisms including small knee flexion angle and large knee internal rotation moment and angle [27].

## Methods

### Participants

Thirty-six healthy participants, including 18 female participants (age 22.1 ± 1.5 years old, height 157.7 ± 6.0 cm, mass 52.5 ± 4.6 kg) and 18 male participants (age 21.8 ± 1.1 years old, height 173.5 ± 5.1 cm, mass 63.6 ± 4.7 kg), were included in the present study. All participants had engaged in recreational sports such as soccer, tennis, or lacrosse at least three times per week for a minimum of 30 min each session. Participants were excluded from this study if they reported a history of knee injury including ACL tear, lower extremity or trunk surgery, neuromuscular disorders, or musculoskeletal injuries within the previous 6 months. All participants read and signed informed consent forms before they were included in this study. The present study was approved by the review board of our institute.

### Procedures and data collection

All data were collected with a Cortex 5.0.1 motion analysis system (Motion Analysis Corp., Santa Rosa, CA, USA). Using this analysis system, synchronized marker coordinate data and force data were recorded with six high-speed cameras (Hawk cameras, Motion Analysis Corp.) and a force plate (Type 9286, Kistler AG, Winterthur, Switzerland). Marker coordinate data and force data were sampled at 200 Hz and 1000 Hz, respectively.

**Fig. 1:**
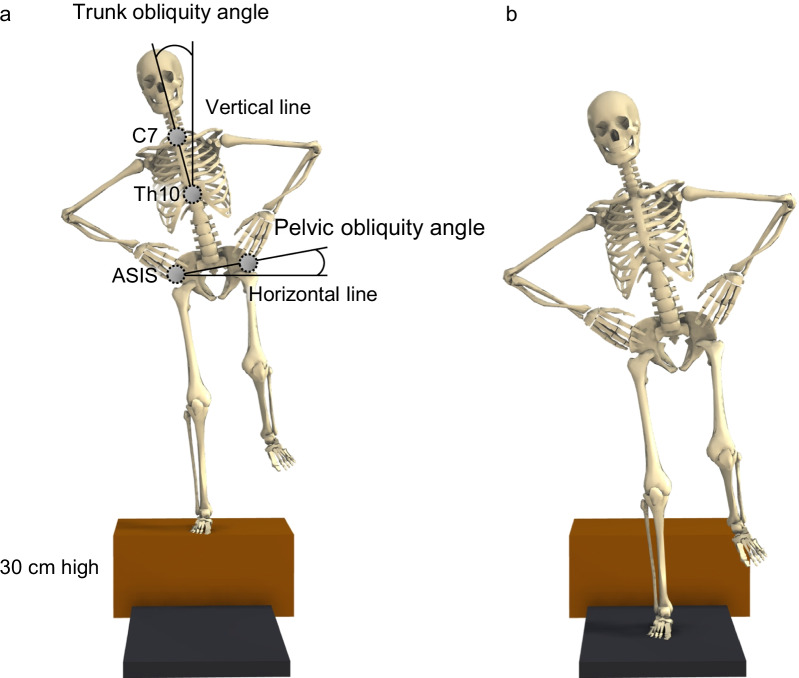
Single-leg landing task. Participants stood on a 30-cm high box (**a**), then dropped off of the box and landed on a force plate that was on the same side as the landing leg (**b**). In the trunk-obliquity landing condition, participants were asked to incline their trunk by 15° toward the landing leg while on the box and keep this orientation until the landing foot contacted the force plate. Under natural landing condition, no specific instructions about trunk position were given. The trunk obliquity angle was defined as the angle between the line connecting C7 and the Th10 marker and a vertical line

The right legs of all participants were analyzed since that was the dominant leg for all participants. The dominant leg was defined as the side preferable for kicking a ball. A total of 41 retroreflective markers were placed on the spinous processes of 7th cervical spinous process (C7) and 10th thoracic spinous process (Th10), the sacrum, the right iliac crest and the medial knee as well as on the participants’ left and right shoulders, anterosuperior iliac spine (ASIS), greater trochanter, hips, lateral knees, medial and lateral malleoli, heels, and second and fifth metatarsal heads; also, cluster markers were placed on the participants’ right thigh and shank [28]. First, data were recorded from participants while they were standing still so that scaling could be performed for each participant. Then, all participants performed single-leg landing from a 30-cm high box under two conditions as follows: (1) the participants performed single-leg landing without any instructions about trunk position (natural landing), and (2) the participants landed with their trunk leaning laterally (trunk-obliquity landing). In the trunk-obliquity condition, participants stood with their right leg on the box, and they inclined their trunk to the right by 15° (Fig. 1a). The angle of the participants’ laterally inclined trunk was measured using a goniometer by a single researcher (S.T.). The angle of a laterally inclined trunk was defined as being formed by the line between the markers of C7 and Th10 and the vertical line on the frontal plane [29]. The lateral incline of the trunk was determined based on the data of previous video-analysis studies on ACL injuries [18, 19]. Participants were asked to drop off the box and land with their right leg on the force plate while maintaining their trunk inclination (Fig. 1b). If the trunk inclination angle obviously changed before the initial contact (IC), the trial was considered a failure, and another trial was conducted. Participants placed their hands on their iliac crests under both landing conditions. Participants were allowed to perform practice trials until they felt familiar with each landing condition. Three successful trials were recorded under each condition. A trial was considered successful if the participant was able to stand still for at least 3 s after landing.

### Data processing and reduction

Kinematic data were low-pass filtered using a 4th order 12 Hz Butterworth filter. The kinematics of the knee and hip joint were calculated using a joint coordinate system with Visual3D software (C-Motion Inc., Germantown, MD, USA). Zero-references were set at the knee and hip angles during the static standing trial [30]. In addition, the angle the participants’ laterally inclined trunk and pelvis were calculated using a custom MATLAB program (MathWorks, Inc., Natick, MA, USA). The angle of the laterally inclined trunk was defined as the angle between the lines connecting the C7 and Th10 markers and the vertical line on the frontal plane [29]. The angle of a laterally inclined pelvis was defined as the angle between the line connecting both ASIS markers and a horizontal line on the frontal plane (Fig. 1a) [9]. The angles of the laterally inclined trunk and pelvis were calculated separately as the angle to the global coordinate system. Force plate data were low-pass filtered using a 4th-order Butterworth filter at 50 Hz [31]. External moments of the knee and hip joints were calculated using inverse dynamics with Visual3D software. Anthropometric properties were based on a previous report [32]. IC to the ground was defined as the point when the vertical ground reaction force (VGRF) exceeded 10 N [30]. The landing phase was defined from the IC to a time point that was double the time from IC to the peak VGRF [12]; this phase was evaluated because this phase places an athlete at greater risk of ACL injuries [33]. Knee and hip joint angles in three planes at the IC were measured. In addition, the peak value of knee and hip joint angles and moments in three planes as well as the peak VGRF during the landing phase were derived. Positive joint angles indicated knee flexion, knee abduction, knee internal rotation, hip flexion, hip adduction, hip internal rotation, lateral trunk obliquity toward the landing leg, and pelvic elevation on the side of the contralateral leg. Positive external moments indicated knee flexion, knee abduction, knee internal rotation, hip flexion, hip abduction, and hip internal rotation. External moments were normalized to each participant’s height and body mass (Nm/m/kg). The VGRF was normalized to each participant’s body mass (N/kg).

### Statistical analysis

Although no study has examined the interaction effect between lateral trunk obliquity and sex on frontal plane knee and hip moments and angles, the effect sizes of lateral trunk obliquity on knee abduction moment and angle were reported to be medium to large [12, 14]. Therefore, we assumed a medium effect size for the interaction effect between trunk obliquity and sex. A total of 34 participants were enrolled to detect a medium effect size (F) of 0.25 with a significance (α) and statistical power (1 − β) of 0.05 and 0.80, respectively. Repeated measures two-way analysis of variance (ANOVA) was conducted with sex as a between-participant factor and landing conditions as a within-participant factor for each dependent variable. The dependent variables included the peak moments of the knee and hip joints in three planes and the peak VGRF during the landing phase. In addition, the dependent variables also included the angles at the IC and the peak angles during the landing phase for the inclined trunk and pelvis and for the knee and hip joints in three planes. Bonferroni post hoc analyses were performed. All data were analyzed with IBM SPSS Statistics 22 (IBM, Armonk, NY, USA). Statistical significance was set at *P* < 0.05. In addition, Cohen’s *d* was calculated as the effect size for each pairwise comparison. Cohen’s *d* was interpreted as follows: *d* > 0.80 large, 0.80 > *d* > 0.50 medium, and 0.50 > *d* > 0.20 small [34].

## Results

The trunk obliquity angle at the IC was 14.3 ± 3.2° and 15.8 ± 3.8° in the trunk obliquity landing for female and male participants, respectively (Table 1).There was no sex difference in the trunk obliquity angle at IC (*P* = 0.555, *d* = 0.08), whereas the trunk-obliquity condition exhibited a significantly larger lateral trunk obliquity angle at the IC than the natural condition (*P* < 0.001, 95% CI 9.9 to 12.2°, *d* = 3.63). The peak trunk obliquity angle was also significantly larger in the trunk-obliquity landing condition than in the natural landing condition (*P* < 0.001, 95% CI 9.7 to 12.3°, *d* = 3.46), while there was no sex difference in the peak trunk-obliquity angle (Table 1). The pelvic obliquity angle was also significantly larger in trunk-obliquity landing than in natural landing (at IC: *P* < 0.001, 95% CI 1.2° to 2.5°, *d* = 0.58; peak angle: *P* < 0.001, 95% CI 1.4° to 2.6°, *d* = 0.67) (Table 1). There was no interaction or sex effect on the pelvic obliquity angle.

**Table 1:** Comparison of trunk and pelvic angles (degrees)

	Female		Male		*P* value		
	Natural	Trunk obliquity	Natural	Trunk obliquity	Landing condition	Sex	Interaction
At initial contact							
Lateral trunk obliquity	4.2 ± 2.0	14.3 ± 3.3	3.8 ± 2.5	15.8 ± 3.9	** < 0.001**	0.555	0.113
Lateral pelvic obliquity	11.6 ± 3.2	13.2 ± 3.3	9.8 ± 3.0	11.9 ± 3.3	** < 0.001**	0.149	0.471
Peak angle							
Lateral trunk obliquity	5.6 ± 2.0	15.7 ± 3.4	5.9 ± 3.3	17.7 ± 3.6	** < 0.001**	0.182	0.156
Lateral pelvic obliquity	12.3 ± 3.1	14.0 ± 3.1	10.3 ± 2.7	12.7 ± 2.9	** < 0.001**	0.087	0.289

Mean ± SD

Bold values indicate a significant effect in two-way repeated-measures ANOVA

**Fig. 2:**
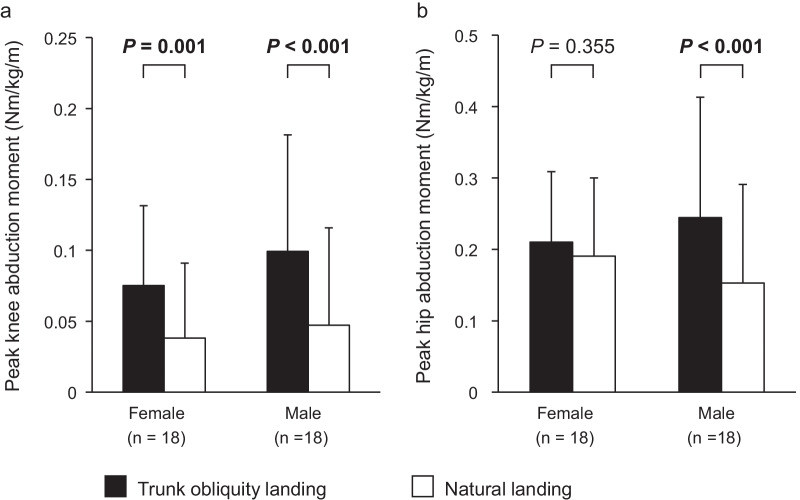
Comparison of peak external knee abduction moment and hip abduction moment between the two single-leg landing conditions. A significant main effect of trunk obliquity was found for peak knee abduction moment (*P* < 0.001) (**a**), while significant effects of landing condition and a sex-by-landing condition interaction were found for peak hip abduction moment (*P* < 0.001 and *P* = 0.021) (**b**). *P* values indicate the results of the post hoc comparison between trunk-obliquity landing and natural landing condition

A significant main effect of trunk obliquity was found for the peak knee abduction moment (*P* < 0.001), and there was no sex or interaction effect on the peak knee abduction moment (Table 2). Trunk-obliquity landing resulted in a significantly larger knee abduction moment than natural landing (95% CI 0.031 to 0.059 Nm/kg/m, *d* = 0.66) (Fig. 2a). On the other hand, significant interaction and trunk-obliquity effects were found for hip abduction moment (*P* = 0.021 and *P* < 0.001) (Table 2). In addition, male participants had increased peak hip abduction moment in the trunk-obliquity landing condition compared with that in the natural landing condition (*P* < 0.001, 95% CI 0.049 to 0.134 Nm/kg/m, *d* = 0.58) (Fig. 2b). On the other hand, female participants showed no difference in hip abduction moment between the trunk-obliquity and natural landings (*P* = 0.355, 95% CI − 0.23 to 0.062 Nm/kg/m, *d* = 0.18). Significant trunk obliquity effects were also revealed for hip flexion and knee flexion moments (*P* = 0.041 and *P* = 0.009) (Table 2). Participants exhibited significantly larger hip and knee flexion moments in the trunk-obliquity landing condition than in the natural landing condition (hip flexion moment: 95% CI 0.005 to 0.203 Nm/kg/m, *d* = 0.18; knee flexion moment: 95% CI 0.017 to 0.111 Nm/kg/m, *d* = 0.26). A sex effect was found for the peak VGRF, and female participants showed significantly smaller peak VGRF than male participants (*P* = 0.036, 95% CI 0.3 to 7.2 N/kg, *d* = 0.71).

**Table 2:** Comparison of the peak hip and knee joint moments and peak vertical ground reaction force

	Female		Male		*P* value		
	Natural	Trunk obliquity	Natural	Trunk obliquity	Landing condition	Sex	Interaction
Peak external moment (Nm/kg/m)							
Hip flexion	1.79 ± 0.50	1.90 ± 0.65	1.96 ± 0.58	2.06 ± 0.54	**0.041**	0.387	0.879
Hip abduction	0.19 ± 0.11	0.21 ± 0.10	0.15 ± 0.14	0.24 ± 0.17	**0.001**	0.968	**0.021**
Hip internal rotation	0.06 ± 0.07	0.07 ± 0.06	0.08 ± 0.06	0.10 ± 0.07	**0.025**	0.267	0.512
Knee flexion	1.82 ± 0.24	1.90 ± 0.21	1.78 ± 0.27	1.82 ± 0.25	**0.009**	0.483	0.480
Knee abduction	0.04 ± 0.05	0.08 ± 0.06	0.05 ± 0.07	0.10 ± 0.08	** < 0.001**	0.445	0.279
Knee internal rotation	0.14 ± 0.06	0.11 ± 0.06	0.16 ± 0.06	0.13 ± 0.07	** < 0.001**	0.397	0.532
Peak vertical ground reaction force (N/kg)	37.6 ± 5.2	37.7 ± 5.3	41.6 ± 5.2	41.2 ± 5.1	0.731	**0.036**	0.580

Mean ± SD

Bold values indicate a significant effect in two-way repeated-measures ANOVA

There was no interaction effect on hip and knee joint angles (Table 3). Trunk obliquity effects were found on the hip adduction angle at the IC, peak hip adduction angle, peak knee flexion angle, and peak knee abduction angle. The peak knee abduction angle was significantly larger in trunk-obliquity landing than in natural landing (*P* = 0.005, 95% CI 0.2° to 0.8°, *d* = 0.19). Participants landed with smaller hip adduction angle at IC (more hip abduction position) and smaller peak hip adduction angle under trunk-obliquity condition than under natural condition (at IC: 95% CI 1.8° to 3.1°, *d* = 0.69; peak angle: 95% CI 2.1° to 4.0°, *d* = 0.64). In addition, participants demonstrated significantly larger peak knee flexion angle in the trunk-obliquity landing condition than in the natural condition (*P* < 0.001, 95% CI 0.7° to 2.3°, *d* = 0.22). Sex effects were found on the hip internal rotation angle at the IC, peak hip flexion angle, and peak hip internal rotation angle. Female participants showed a larger peak hip flexion angle than male participants (*P* = 0.010, 95% CI 1.3° to 9.2°, *d* = 0.89). Additionally, female participants landed with smaller hip internal rotation angle at the IC (more hip external rotation position) and smaller peak hip internal rotation angle (at the IC: 95% CI 2.0° to 9.3°, *d* = − 1.0; peak angle: 95% CI 0.9° to 7.6°, *d* = − 0.86).

**Table 3:** Comparison of knee and hip joint angles

	Female		Male		*P* value		
	Natural	Trunk obliquity	Natural	Trunk obliquity	Landing condition	Sex	Interaction
Angle at IC (°)							
Hip flexion	22.4 ± 4.6	21.7 ± 5.1	18.8 ± 4.9	19.2 ± 4.5	0.663	0.059	0.059
Hip adduction	− 5.9 ± 3.6	− 8.1 ± 3.7	− 4.2 ± 3.3	− 6.9 ± 3.4	** < 0.001**	0.210	0.503
Hip internal rotation	− 8.3 ± 5.5	− 8.1 ± 4.9	− 3.3 ± 5.8	− 1.8 ± 6.0	0.072	**0.004**	0.178
Knee flexion	18.4 ± 5.5	19.2 ± 6.0	16.5 ± 5.3	17.2 ± 5.8	0.055	0.292	0.817
Knee abduction	− 2.0 ± 2.0	− 1.6 ± 2.0	− 1.4 ± 1.6	− 1.3 ± 1.6	0.065	0.510	0.271
Knee internal rotation	− 7.1 ± 4.3	− 6.8 ± 3.8	− 6.9 ± 4.6	− 6.9 ± 4.7	0.680	0.978	0.774
Peak angle (°)							
Hip flexion	36.9 ± 6.0	36.9 ± 6.3	31.0 ± 6.0	32.3 ± 5.3	0.163	**0.010**	0.134
Hip adduction	7.2 ± 4.7	4.4 ± 6.1	5.3 ± 3.6	2.0 ± 4.1	** < 0.001**	0.163	0.603
Hip internal rotation	0.6 ± 5.5	0.8 ± 5.2	4.5 ± 4.6	5.4 ± 4.8	0.145	**0.014**	0.324
Knee flexion	57.4 ± 8.4	59.1 ± 8.2	53.5 ± 4.9	55.0 ± 5.1	** < 0.001**	0.084	0.822
Knee abduction	0.1 ± 2.8	0.5 ± 2.6	− 0.4 ± 2.6	0.1 ± 2.7	**0.005**	0.581	0.593
Knee internal rotation	4.0 ± 5.2	4.1 ± 5.5	3.3 ± 5.5	3.7 ± 5.5	0.355	0.753	0.448

Mean ± SD

IC, initial contact

Bold values indicate a significant effect in two-way repeated-measures ANOVA

## Discussion

The purpose of the present study was to compare the effects of the laterally inclined trunk on knee abduction moment as well as knee and hip kinetics and kinematics between female and male participants during single-leg landing. The main finding of the present study was a significant interaction effect between trunk obliquity and sex on the peak hip abduction moment. To our knowledge, this is the first study showing that the effect of trunk obliquity on frontal plane hip moment differed between females and males. Although lateral trunk obliquity toward the side of the landing leg increased peak knee abduction moment, we failed to find a sex-based difference in the effects of the intentional inclined trunk on peak knee abduction moment and knee abduction angle. Therefore, the present findings partially support our hypothesis.

Under the trunk-obliquity condition, the participants were asked to maintain the angle of their laterally inclined trunk at 15° until the IC. Female and male participants showed 14.3° and 15.8° lateral trunk obliquity angle at the IC under the trunk-obliquity condition, and there was no sex difference in the angle of the laterally inclined trunk at IC. Trunk and pelvic obliquity angles increased under trunk obliquity condition compared with those under natural landing condition. The increase in trunk obliquity angle at IC was 9.9 to 12.2° with a 95% CI, while the increase in pelvic obliquity angle was 1.2° to 2.5° with a 95% CI. Therefore, the changes in hip and knee angles and moments would be mainly caused by lateral trunk obliquity. Additionally, the peak knee abduction moment was significantly increased under the trunk-obliquity condition compared with that under the natural condition, which is consistent with the findings reported in previous studies [10, 12]. The landing task with lateral trunk obliquity was properly performed, allowing a comparison of the effect of trunk obliquity on knee and hip kinetics and kinematics between females and males.

The effect of lateral trunk obliquity on knee and hip moments and angles was similar for male and female participants except for hip abduction moment. The hypothesis that female participants would show a greater increase in knee abduction moment due to lateral trunk obliquity than male participants was not supported. This hypothesis was motivated by previous theories that lateral trunk obliquity is a female-specific ACL injury mechanism [11, 35]. The present findings suggest that one of the reasons why only female athletes demonstrated lateral trunk obliquity in video analysis studies of ACL injuries may involve the fact that female athletes are more likely to incline their trunk during sports [18–21]. Lateral trunk stability in response to external loading is a predictor of knee injuries including ACL tears [36]. However, well-controlled motion analysis studies including the present study have not detected sex differences in trunk obliquity angle during landing [37, 38]. Therefore, it is not well understood whether female athletes are prone to lateral trunk incline. Future studies should investigate sex differences in trunk control during more sports-like landing tasks.

Male participants had increased peak hip abduction moment in response to a laterally inclined trunk. This change is consistent with a previous finding showing that the lateral trunk obliquity angle was significantly correlated with the hip abduction moment during a lateral reactive jump [8]. The increase in hip abduction moment suggests increased activity of the hip adductor muscles. Pertinently, the hip adductor muscles control pelvic-on-femoral hip movement in the frontal plane [39]. During single-leg activity, the hip adductor muscles are considered to control the trunk via the pelvis in response to changes in pelvic position relative to the femur [26]. Male participants counteract increasing reactive hip adductor activities to stabilize their trunk and pelvis under trunk-obliquity condition [11]. On the other hand, female participants showed no change in peak hip abduction moment between the natural and trunk-obliquity conditions. These findings suggest the possibility that female individuals have poor neuromuscular control of the hip joint in the frontal plane in response to a laterally inclined trunk. However, the present study failed to detect an interaction effect on the peak trunk and knee angles or moments including the knee abduction moment. One possible reason for this lack of an interaction effect may be that the trunk obliquity in the present study was intentional and under well controlled conditions. Therefore, future study studies are needed to clarify sex differences in hip and knee kinetics and kinematics in response to lateral trunk obliquity in a setting closer to actual sport situations, such as lateral trunk obliquity with ball catching [12] or with trunk rotation [40].

In both male and female participants, lateral trunk obliquity increased peak knee abduction moment compared with those in the natural landing condition, which is consistent with previous findings [9, 10, 12]. This is a rationale for avoiding lateral trunk obliquity to prevent ACL injuries because the knee abduction moment is one ACL injury mechanism [9, 22–24]. Although the peak knee abduction angle was also increased under trunk obliquity condition, it should be noted that the 95% CI of the difference between the two conditions was less than 1°. Peak hip flexion moment, peak knee flexion moment and peak knee flexion angle increased under the trunk-obliquity condition compared with the measures observed in the natural condition. These changes indicate that participants attempted to have softer landings under the trunk-obliquity condition [41]. A soft-landing strategy was associated with a small knee abduction moment and knee abduction angle during landing [42]. The change in the peak knee abduction angle due to lateral trunk obliquity may be small in the present study because participants used a soft-landing strategy. However, it should be noted that the peak knee abduction moment was significantly larger under the trunk-obliquity condition despite the soft landing observed. These findings indicate that the control of lateral trunk obliquity is more important than a soft landing in the effort to reduce the knee abduction moment. The peak knee internal rotation moment also decreased under the trunk-obliquity condition, which is in agreement with previous studies [10, 12]. A recent study reported associations between smaller knee internal rotation motion and larger hip flexion motion or larger knee flexion moment during landing [43]. The findings of the present and previous study suggest associations of smaller knee rotation motion and moment with soft landing. However, since there are few reports on the association of a soft landing with knee rotation moment and motion, further research is necessary to clarify these relationships.

Concerning clinical relevance, lateral trunk obliquity increased peak knee abduction moment despite soft landing for both male and female participants. Therefore, as in previous theories [9, 22–24], jump-landing training to avoid lateral trunk obliquity during landing is important to prevent ACL injury. In addition, female participants did not change their hip abduction moment in response to the lateral inclination of their trunk during single-leg landing, while male participants increased their hip abduction moment. Although hip abductor muscles have been a focus in prevention training for ACL injuries [23, 44], hip adductors are also important to control pelvis and trunk inclination [11, 26, 39]. The present study suggests that female participants have poor control of their hip joint in response to the inclination of their trunk, which may lead to larger motion of their pelvis and trunk in the frontal plane during sports maneuvers. Previous laboratory studies showed that female individuals showed a smaller inclination of their pelvis and trunk toward a support leg during a change-in-direction task than males demonstrated [25, 45]; hence, females may avoid pelvic and trunk obliquity due to poor control of the hip joint. The present findings suggest that it would be beneficial to focus on frontal plane hip joint control under lateral trunk obliquity during single-leg landing for the prevention of ACL injury in female individuals. Further studies are needed to reveal whether hip adductor training improves pelvic and trunk control during athletic tasks.

Some limitations should be acknowledged. First, lateral trunk obliquity in the present study was intentional, and landing was performed in a well-controlled condition. The participants performed single-leg landings while maintaining their trunk inclination until the IC and placing their hands on their iliac crests. In actual sports situations, the effect of lateral trunk obliquity on knee and hip kinetics and kinematics may differ between female and male athletes. Further studies should be conducted to determine sex differences in hip and knee joints in response to trunk obliquity during landing in more sports-like settings. Second, we did not measure any muscle activity, although we expected that male participants increased hip adductor activity and that but female participants did not based on the results of the interaction on the hip abduction moment. Future studies should be conducted to evaluate sex differences in muscle activity. Third, the lateral flexion of the spine was not controlled in the present study. Although there was no sex effect of the interaction between sex and landing condition on the trunk obliquity angle or pelvic obliquity angle, the difference in the lateral flexion angle of the spine of participants may have affected the results of the present study. Finally, we investigated the effects of lateral trunk obliquity, sex and their interaction on knee and hip moments and angles not only in the frontal plane but also in the sagittal and horizontal planes because ACL injuries are caused by a multiplanar loading mechanism [27]. Other studies used similar statistical comparisons of hip and knee kinetics and kinematics on three planes with a similar study design [46–48]. Although we consider that this method is acceptable, we should acknowledge that the number of statistical comparisons affects the study-wise type I error rate.

## Conclusions

Knee abduction moment increased with a laterally inclined trunk for both female and male participants without a sex difference. In addition, male participants increased hip abduction moment in response to a lateral inclination of their trunk, while female participants did not exhibit a change in their hip abduction moment. The present findings suggest that female participants’ control of their frontal plane hip joint did not change in response to a laterally inclined trunk. Therefore, in addition to jump landing training to avoid lateral trunk obliquity, it would be beneficial to focus on frontal plane hip joint control under lateral trunk-obliquity conditions during jump landing for the prevention of ACL injuries in female individuals.

## Data Availability

The datasets used and analyzed during the current study are available from the corresponding author upon reasonable request.

## Abbreviations

ACL: Anterior cruciate ligament
IC: Initial contact
VGRF: Vertical ground reaction force
C7: 7th cervical spinous process
Th10: 10th thoracic spinous process
ASIS: Anterosuperior iliac spine
ANOVA: Analysis of variance
